# Analysis of the Power of Common Diagnostic Tools in the Management of Acute Pancreatitis

**DOI:** 10.1155/2014/438697

**Published:** 2014-08-20

**Authors:** Markus Nistal, Malai Zoltani, Ansgar W. Lohse, Nicola Di Daniele, Manfredi Tesauro, Andrea Pace

**Affiliations:** ^1^Sozialstiftung Bamberg-Medizinische Klinik II, 96049 Bamberg, Germany; ^2^Medizinische Klinik I, Universitaetsklinikum Hamburg-Eppendorf, 20246 Hamburg, Germany; ^3^Department of Internal Medicine, University of Rome Tor Vergata, Viale Oxford 81, 00133 Rome, Italy; ^4^Klinik für Gastroenterologie, Friedrich-Ebert-Krankenhaus, 24534 Neumünster, Germany

## Abstract

Acute pancreatitis (AP) is a serious medical condition usually associated with severe upper abdominal pain. The purpose of our study is to assess the therapeutic consequences of contrast-enhanced computed tomography (CE-CT) and the predictive value of CRP for severe pancreatitis. We included patients with a threefold increase of plasma lipase who had received a CE-CT or had a CRP of =150 mg/dl. A total of 74 out of 283 patients got a contrast-enhanced CT scan; in 11 cases the CT was followed by endoscopic or surgical interventions as therapeutic consequences compared with 19 out of 50 control cases. 69 out of 283 patients (24,3%) had CRP >150 mg/dl within 48 hours after admission. 32 of them had SAP. The CRP cutoff of 150 mg/L had a sensitivity of 80% and a specificity of 65%. The positive predictive value for SAP in patients beyond the cutoff is 46.4%. The negative predictive value for SAP in patients below the cutoff was 89.5%. Our results support the opinion that an early CE-CT is usually not indicated. CRP helps to assess the course of AP; levels below 150 mg/dl between the first 48 h indicate a mild course in most of the cases.

## 1. Background

Acute pancreatitis (AP) is a common but complex condition with many difficulties in clinical practice. Despite the implementation of various classification criteria and national and international guidelines there is still insecurity in the assessment of severity of AP as well as in the choice of specific therapy for this disease. In Germany the incidence of acute pancreatitis has been determined at about 16.0/100,000 in men and 10.2/100,000 in women and seems to be constant over the past years [1]. Causal factors are mostly gallstones, followed by alcohol abuse, a relatively high number of approximately 20% cases of undetermined etiology (“idiopathic”), and miscellaneous etiologies summing up to about 10% of the cases [1].

The categorization of AP has long been based on the Atlanta classification of 1992, which classifies cases with systemic complications such as organ failure and/or local complications like infected necrosis as severe acute pancreatitis (SAP) [2]. These cases account for about 15% overall, while 85% have an uncomplicated course without systemic or local complications and are classified as interstitial, oedematous, mild, or nonsevere acute pancreatitis [3]. The definitions of the Atlanta classification have often been found confusing [4]. This together with new insights concerning prognostic differences in several patient subgroups has led to a new international classification of acute pancreatitis, which distinguishes three grades of severity [5]. Following the current diagnostic criteria for the diagnosis of AP, two of the following three criteria are required: (A) abdominal pain consistent with AP, (B) serum lipase activity over three times than the upper limit of normal, and (C) characteristic findings on contrast-enhanced computed tomography (CE-CT) [3, 5, 6]. Typical findings for nonsevere pancreatitis are oedematous swelling of the organ and a homogenous contrast enhancement; severe forms of AP can show noncirculated, necrotic areas, and fluid collections intra- and extrapancreatic [3].

There is evidence for the indication of an ultrasound examination to detect biliary etiology and indicate endoscopic retrograde cholangiopancreaticography (ERCP); the role and the correct timing of CE-CT remains a controversial issue [6]. A major indication for CE-CT is detection of pancreatic and peripancreatic complications during the course of AP. Pancreatic necrosis can be visualized usually within 96 h, but not within the first 48 h after onset of symptoms [7]. Clinically relevant collections of pancreatic juice (pseudocysts) take at least four weeks to develop [7]. Looking at the value of CE-CT in predicting SAP and mortality, it does not exceed the power of several clinical scoring systems used on predicting the severity of acute pancreatitis [8]. Up to now the Acute Physiology and Chronic Health Evaluation (APACHE) II-score has been considered the most reliable system [9]. A score higher than 8 is associated with a severe course with higher mortality, although predictive values show the limitations for this complex scoring system [3]. There is an ongoing search for a simple, broadly available diagnostic tool. C-reactive protein (CRP) is a routine parameter reflecting inflammative processes of any etiology. A cutoff of 150 mg/dL has been established as an independent prognostic factor for SAP [10], which is broadly used to determine severity of pancreatitis.

Our goal is to assess the therapeutic consequences of contrast-enhanced computed tomography and the predictive value of CRP for severe pancreatitis in a retrospective analysis of clinical data (Table 1). Furthermore we intended to determine the occurrence of acute abdomen in clinical examination of patients with severe and nonsevere AP compared to the occurrence of CE-CT findings typical for AP.

## 2. Methods

We generated a list of cases with a main diagnosis of acute pancreatitis of any cause from our accounting department covering a span from July 2008 to June 2011. A total number of 283 cases were reviewed by extracting data from Soarian Clinicals, the electronic file used at the university medical center at Hamburg-Eppendorf. We identified all cases with a threefold increase of plasma lipase (>180 U/l) and complete availability of diagnostic procedures, lab results, and discharge reports. Doubtful cases, for example, patients with exacerbations of chronic pancreatitis but without significant enzyme evaluation, were excluded. Also, patients with insufficient data available, for example, due to referral from another facility, were excluded. From the remaining cases all patients who had received a CE-CT or had a CRP of ≥150 mg/dL were included. Control cases were extracted from the remaining identified cases at random. Altogether a total number of 154 was used for the following examinations and is referred to as “study group.”

### 2.1. Assessing Therapeutic Consequences of Contrast-Enhanced Computed Tomography

We identified all CT examinations from our study group by browsing files and reports. Results were divided into CTs performed within the first 48 hours after admission and scans after 48 hours of admission. The group within 48 hours was matched with a same size group of cases without CT within the first 48 hours. For all three groups we assessed occurrence of therapeutic consequence beyond basic management of pancreatitis after imaging. As therapeutic consequences we defined endoscopic and surgical procedures affecting the pancreaticobiliary system: (a) endoscopic retrograde cholangiopancreaticography (ERCP), (b) endoscopic debridement of necrosis or drainage of pseudocysts, or (c) open or laparoscopic debridement of necrosis or drainage of pseudocysts.

### 2.2. Assessing Predictive Value of CRP on Severity of Pancreatitis

All cases with a CRP >150 mg/L, within the first 48 hours, were identified from our study group by browsing the lab results. The identified group of cases was matched with a group of patients with a CRP <150 mg/dL. Both groups were examined for development of severe pancreatitis during the clinical course. According to Atlanta classification we defined cases with severe pancreatitis by occurrence of local or systemic complications. Local complications were defined as necrosis, abscesses, or pseudocysts identified on a CT scan. Systemic complications were considered shock (systolic blood pressure <90 mmg) and pulmonal insufficiency (PaO2 < 60 mmHg). Renal failure was defined as serum creatinine ≥2 mg/dL after 24 h of fluid resuscitation. In patients with underlying chronic kidney failure we defined an increase of ≥1 mg/dL above baseline creatinine (lowest creatinine during stay) as acute renal failure due to AP. Coagulation disorders (platelets ≤ 100.000/*µ*L, fibrinogen < 1.0 g/L) and severe metabolic disturbances (calcium ≤ 1.87 mmol/L) were defined as systemic complications too, following Atlanta classification.

### 2.3. Diagnostic Value of Clinical Examination versus CE-CT on Admission

We took the identified cases with SAP from assessing the predictive value of CRP and checked how many of them had symptoms of acute abdomen, which were defined as abdominal pain during palpation and/or guarding. The number of them, who were examined with a CE-CT and had findings fitting with acute pancreatitis, was determined as well. The cases of SAP were matched to the population with mild pancreatitis identified from assessing the predictive value of CRP. We compared the occurrence of abdominal findings of acute abdomen with the occurrence of pathologic findings on CE-CT in both groups.

## 3. Results

We identified a total of 154 patients for the study group. Their average age was 49 years; 33.8% were feminine and 66.2% were masculine. 35.7% of them had a biliary etiology, and 28.6% were caused by ongoing alcohol abuse. The remaining cases include idiopathic and miscellaneous etiology and several cases with exacerbation of underlying chronic pancreatitis with significantly elevated enzyme levels to diagnose AP.

### 3.1. Assessing Therapeutic Consequences of Contrast-Enhanced Computed Tomography

We identified a total of 74 out of 283 patients who got a contrast-enhanced computed tomography during their stay. 50 of them received their scan within 48 hours after admittance. In 11 cases (22%) the CT was followed by endoscopic or surgical interventions as therapeutic consequences compared with 19 out of 50 control cases (38%). In patients with a CT beyond 48 hours after admittance, in 6 out of 24 (25%) cases therapeutic consequences occurred after the scan.

### 3.2. Assessing Predictive Value of CRP on Severity of Pancreatitis

69 out of 283 patients (24,3%) had CRP >150 mg/dL within 48 hours after admission. 32 of them (46.4%) had SAP following Atlanta classification. In a control group with 76 patients 8 of them (10.5%) had a severe course. The CRP cutoff of 150 mg/L had a sensitivity of 80% and a specificity of 65%. The positive predictive value for SAP in patients beyond the cutoff is 46.4%. The negative predictive value for SAP in patients below the cutoff was 89.5%.

### 3.3. Diagnostic Value of Clinical Examination versus CE-CT on Admission

During assessing predictive value of CRP we identified 40 cases of AP with severe course. 37 of them (92.5%) had a presentation of acute abdomen in clinical examination. 31 of them received CE-CT, and 30 (96.7%) of them had significant findings for AP.

Within the control group we checked on 115 patients with mild AP. 93 (80.9%) of them had an acute abdomen in clinical examination. 24 received CE-CT, of which 19 (79.2%) had significant alterations fitting to AP.

## 4. Discussion

Despite the known low diagnostic value of early CT scans between the first 48 hours after admission, approximately two of three scans were performed during that time window. Therapeutic consequences occurred in a lower percentage of cases compared to a control group without CE-CT. The higher percentage of interventions in the control group is probably due to many cases of biliary pancreatitis that received ERCP after ultrasound examination compared with a higher number of cases with alcoholic pancreatitis that received CT without any consequences. The results support the current opinion that an early CE-CT is usually not indicated and should be restricted to the rare situation of clinical uncertainty. CE-CT might have be used to ensure to the physician that no precarious differential diagnoses may have been overlooked. In consideration of the radiation load, costs, and possible severe complications like worsening of renal insufficiency due to contrast agent, we believe this practice to not be reasonable. In cases with CE-CT after the first 48 hours the percentage with therapeutic consequences was higher, as to be expected, but it only occurred in one of four cases. This supports the hypothesis that detection of local complications in most cases does not lead to interventional or operative steps. However, CE-CT remains the crucial technique to plan interventions in patients worsening due to infected necrosis or pancreatic abscess.

Compared to available data with a sensitivity of 80% the determined sensitivity is in range of the published data [11]. Specificity has been determined lower than the previously reported 76% [11]. The positive predictive value of the examined CRP cutoff can be stated as low, so from our data a severe course of AP could not be predicted with the certainty needed. The negative predictive value of the cutoff is acceptable. We may conclude that in our cases severe courses could be ruled out with an acceptable probability. Our data supports the conception to use CRP as an easily accessible marker to determine the risk for a severe course. Since CRP correlates well with the presence of local complications such as necrosis [12], another tool to identify organ failure easily might be useful to add to compensate the low positive predictive value. Following the recommendations of the new classification [5], daily appliance of the Modified Marshall scoring system for organ dysfunction, especially in patients with a CRP above the cutoff, could be a good second cornerstone to distinguish between mild and severe cases in the early phase objectively besides the important but necessarily subjective clinical examination. In our opinion, given the high negative predictive value of the cutoff, patients with a CRP below the cutoff do not need special precautions or advanced diagnostic tools, since the risk for complications can be considered low.

Our data showed nearly the same occurrence of acute abdomen in clinical presentation and pancreatitis related alterations in CE-CT both in patients with mild AP and SAP. It may be concluded that CE-CT and clinical examination show no distinctive difference in assessing SAP and mild AP in general within admission. To diagnose AP, CE-CT seems to not be more sensitive than clinical examination. This supports the opinion to rely on pancreatic enzymes and clinical presentation to establish the diagnosis of AP by two out of the aforementioned three criteria. CE-CT should be restricted to situations where the diagnosis of AP cannot be made and other differentials of acute abdomen such as mesenterial infarction need to be diagnosed or excluded. Both examinations are more sensitive in cases with SAP. The differences of about 12–15% are not helpful in differentiating between SAP and mild AP.

For all aspects of our study it remains to be stated that, during extraction and assessing our data, Atlanta classification has been the only broadly accepted approach to classify our results. This diminishes the comparability to future studies, which will mostly use the recently published classification with three grades of severity. Cases regarded as severe in our study would have been “moderately severe” or “severe” in the new classification.

## 5. Conclusion

We conclude that CE-CT should be used very restrictively in assessing AP. Especially CTs on or early after admission should not be performed, since clinical examination has almost the same sensitivity. CRP helps to assess the course of AP, and levels below 150 mg/dL between the first 48 h indicate a mild course in most of the cases. A clinical scoring system should be added. We also believe that our findings require confirmation in a prospective study with larger number of patients.

## Figures and Tables

**Table 1:** Assessment of predictive value of CRP on severity of AP.

	CRP > 150 mg/dL	CRP < 150 mg/dL	Total	
Severe course	32	8	40	0.8 (sensitivity)
Mild course	37	68	105	0.65 (specificity)
Total	**69**	**76**		
Predictive values	0.464 (positive)	0.895 (negative)		

## References

[1] Lankisch PG, Karimi M, Bruns A, Maisonneuve P, Lowenfels AB (2009). Temporal trends in incidence and severity of acute pancreatitis in Lüneburg County, Germany: a population-based study. *Pancreatology*.

[2] Bradley EL (1993). A clinically based classification system for acute pancreatitis. *Archives of Surgery*.

[3] Banks PA, Freeman ML, Fass R (2006). Practice guidelines in acute pancreatitis. *The American Journal of Gastroenterology*.

[4] Bollen TL, van Santvoort HC, Besselink MG (2008). The Atlanta classification of acute pancreatitis revisited. *British Journal of Surgery*.

[5] Banks PA, Bollen TL, Dervenis C Classification of acute pancreatitis—2012: revision of the Atlanta classification and definitions by international consensus. *Gut*.

[6] Adler G, Woehrle H (2005). Diagnotik und Therapie der Akuten Pankreatitis. *Internist*.

[7] Bharwani N, Patel S, Prabhudesai S, Fotheringham T, Power N (2011). Acute pancreatitis: the role of imaging in diagnosis and management. *Clinical Radiology*.

[8] Bollen TL, Singh VK, Maurer R (2012). A comparative evaluation of radiologic and clinical scoring systems in the early prediction of severity in acute pancreatitis. *The American Journal of Gastroenterology*.

[9] Brisinda G, Vanella S, Crocco A (2011). Severe acute pancreatitis: advances and insights in assessment of severity and management. *European Journal of Gastroenterology & Hepatology*.

[10] Gonzálvez-Gasch A, García de Casasola G, Barba Martín R, Herreros B, Guijarro C (2009). A simple prognostic score for risk assessment in patients with acute pancreatitis. *European Journal of Internal Medicine*.

[11] Larvin M (1997). Assessment of severity and prognosis in acute pancreatitis. *European Journal of Gastroenterology and Hepatology*.

[12] Schütte K, Malfertheiner P (2008). Markers for predicting severity and progression of acute pancreatitis. *Best Practice and Research in Clinical Gastroenterology*.

