# Corrigendum to “Analysis of the Power of Common Diagnostic Tools in the Management of Acute Pancreatitis”

**DOI:** 10.1155/2018/4929275

**Published:** 2018-06-24

**Authors:** Markus Nistal, Malalai Zoltani, Ansgar W. Lohse, Nicola Di Daniele, Manfredi Tesauro, Andrea Pace

**Affiliations:** ^1^Sozialstiftung Bamberg-Medizinische Klinik II, 96049 Bamberg, Germany; ^2^Medizinische Klinik I, Universitaetsklinikum Hamburg-Eppendorf, 20246 Hamburg, Germany; ^3^Department of Internal Medicine, University of Rome Tor Vergata, Viale Oxford 81, 00133 Rome, Italy; ^4^Klinik für Gastroenterologie, Friedrich-Ebert-Krankenhaus, 24534 Neumünster, Germany

In the article titled “Analysis of the Power of Common Diagnostic Tools in the Management of Acute Pancreatitis” [1], the name of one of the authors was given incorrectly as Malai. The author's name should have been written as Malalai. The revised authors' list is shown above. Additionally, it should have been noted that Markus Nistal and Malalai Zoltani contributed equally to this work as joint first authors of the article.
